# Knowledge, Attitude, and Practice (KAP) of post exposure prophylaxis for fifth year dental students at a private Egyptian university: a cross-sectional study

**DOI:** 10.1186/s12903-023-02890-7

**Published:** 2023-03-24

**Authors:** Hagar Saleem, Nevine Waly, Fatma Abdelgawad

**Affiliations:** Pediatric Dentistry and Dental Public Health, Faculty of Dentistry, Cairo University, Cairo, Egypt

**Keywords:** Post exposure prophylaxis, Dental practitioner, Awareness, HIV, HBV

## Abstract

**Background:**

Health care professionals including dental staff are at greater risk of occupational exposure to life threatened blood-borne pathogens. Occupational exposures will continue to occur despite improved techniques of prevention and using the post exposure prophylaxis (PEP) in these situations are of great importance. Therefore, the aim of this study was to assess the knowledge, attitude, and practice of post exposure prophylaxis for fifth year dental students at a private Egyptian university.

**Methods:**

This cross-sectional study was conducted among 404 dental students in the fifth year at a private Egyptian university from July 2019 to March 2020. Data were collected using self-administrated questionnaires including personal information, knowledge, attitude, and practice questions.

**Results:**

Our study showed that the total mean knowledge score was (0.45 ± 0.50), for attitude (0.70 ± 0.46), and that for practice (0.45 ± 0.50). There was no gender difference regarding PEP (*P* > 0.05). A total of 213 (47.5%) dental students believed that PEP should be indicated for any needlestick injury in the workplace. A number of 379 of the students (94%) chose “Yes” when asked if they thought PEP is important. While, 143 students (32.5%) were unaware of the existence of PEP service and protocol when asked about the reasons for not taking PEP after occupational exposure.

**Conclusions:**

Knowledge and practice of fifth year dental students at a private Egyptian university toward post exposure prophylaxis are not satisfactory. Awareness and curriculum modifications are important regarding PEP.

**Supplementary Information:**

The online version contains supplementary material available at 10.1186/s12903-023-02890-7.

## Background

Healthcare professionals can accidently be introduced to blood or other infectious material while performing their duties due to needlestick injuries (NSI) [1]. It has been reported that, at least 26 different pathogens are transmitted through needlestick and sharp injuries [2, 3]. Annually, according to the World Health Organization (WHO), needlestick injuries affect nearly 3 out of 35 million healthcare workers (HCWs) globally, exposing them to blood-borne pathogens [4, 5].

Occupational exposures will continue to occur despite improved techniques of prevention. Therefore, using the post exposure prophylaxis (PEP) in these situations are mandatory [2]. As a result, HCWs should be aware of the potential risks associated with treatment procedures and take proper measures while dealing with patients [6].

In dentistry, transmission of serious infections can frequently occur as occupational accidents during the usage of local anesthetic syringe, recapping, instruments’ cleaning, and changing the anesthetic carpule. Moreover, during dental treatment, patients may need multiple injections which may increase the risk of injury with contaminated needle [7, 8]. Furthermore, different challenges are faced by the dentists such as restricted field of vision and sudden patients’ movement during the use of needle or sharp instruments. These challenges make the dentist vulnerable to exposure [9].

Dental students represent a category of healthcare professionals that are at great risk for acquiring and spreading infection because they deal closely with blood and other body fluids from the patients in healthcare settings or even laboratories [10]. Dental students are considered the most vulnerable HCWs to blood-borne exposure as they are developing their skills and working in a limited field [11–13]. Although, the risk varies during their career, but it is usually the highest during their professional training [11]. Students face stressful schedules and responsibilities which may lead to less concentration during patient treatment and thus, occupational injuries might occur [8].

According to recent reports by the WHO, occupational exposures cause about 40% of Hepatitis B virus (HBV) and Hepatitis C virus (HCV) worldwide, with Human Immunodeficiency virus (HIV) accounting for 2.5% of infections among HCWs. Correspondingly, chronic cases of HCV, and HBV are also on the rise with over, 200 M chronic HCV carriers, and 350 to 400 M chronic HBV carriers globally [2, 7, 9, 14].

Post exposure prophylaxis is defined by the WHO as the medical intervention offered to prevent the transmission of blood-borne diseases following a potential exposure to HIV, HBsAg, HCV, and other viruses. It should be started as soon as possible, within hours of the probable exposure, and no later than 72 h. The recommended PEP should be started within 6 h of a suspected exposure. It is recommended to start PEP as soon as possible even if the laboratory results did not appear. In most situations, PEP will be immediately stopped after obtaining negative results, avoiding any potential side effects. PEP cannot prevent the infection, but it can limit virus replication by preventing viral DNA from being incorporated into host DNA [9, 15].

Prophylaxis administration shortly after exposure may subside or prevent systemic infection by inhibiting the virus proliferation. When a person is exposed to HIV, systemic infection does not occur immediately, but infection of dendritic cells in the mucosa and skin occurs within the first 24 h at the site of inoculation. Prophylaxis should be administered within the first hour for the maximum benefit, while it can be postponed up to 48 h. After 72 h, PEP is less effective in avoiding infection, although it can still slow the progression of the primary infection [16].

Occupational injuries among HCWs represent a real challenge. It is considered as a significant public health concern. Deficient information was discovered in the literature regarding the awareness level of the dentists and dental students toward post exposure prophylaxis following occupational exposure to the blood-borne viruses. Hence, this study is purposed to assess the knowledge, attitude, and practice of post exposure prophylaxis for fifth year dental students at a private Egyptian university.

## Methods

### Study design

This cross-sectional study was conducted on dental students. These students were registered in the fifth year at October University for Modern Sciences and Arts (MSA) from July 2019 to March 2020. The STROBE guidelines were applied to verify that this observational study was properly reported.

### Sample size and sampling procedure

We performed a pilot study on a previous semester for fifth year dental students. Several questionnaires (51) were retrieved from those students (not included in the final sample of this study) to calculate the sample size. We found that, the calculated sample size from the pilot study was 384 students. The sample size was calculated using the epi info program.

We used a convenience consecutive sampling procedure, where we distributed the questionnaire to all fifth year dental students in their practical sessions.

### Participants

#### Inclusion criteria

• The fifth year dental students studying at MSA university irrespective of age and sex.

#### Exclusion criteria

• Refusal of participation from the students.

### Outcome

This research aimed to assess knowledge, attitude, and practice of fifth year dental students at MSA university toward post exposure prophylaxis.

### Data sources and measurement

A twenty-five item, self-administrated, anonymous structured questionnaire was used in this study. Questions were obtained from a questionnaire that has been pre-validated based on a published article according to Mathewos et al. [17].

Self-administered questionnaire was distributed among fifth year dental students. Questionnaire was distributed once at the end of their clinical sessions and no attempt was made to follow up with the students who were absent on the day of questionnaire distribution. Dental students were asked to participate voluntarily in the study by filling in the questionnaire. Participants were provided with enough time 5–10 min to answer the questions. Before answering the questionnaire, they were provided with a full explanation concerning the aim of the study without guiding them to the answers.

The KAP questionnaire included four sections as follows:

• **Section A (Demographic Data):**

Closed-ended questions on demographic variables were asked. The demographic variables included age and gender.

• **Section B (Knowledge):**

There were nine structured questions in this section. A total of eight questions were used to test general knowledge, with one question assessing PEP's information sources. The knowledge questions evaluated the indications, maximum delay, optimal timing, efficacy, duration of PEP use, training, and PEP recommendations. This section's questions were all closed-ended, with participants being instructed to choose their own responses as needed.

• **Section C (Attitude):**

Seven questions were included in this part to gauge the participants' attitudes regarding PEP for blood-borne viral diseases. The questions centred on the importance of PEP training, the behavioral changes associated with it, providing the work space with formal PEP protocol, the possibility of PEP to prevent further infection, giving PEP for all sharps injuries, and the belief that PEP might be unnecessary in unknown exposure. Negative and positive viewpoints were mixed together to allow for a wide spectrum of attitudes to be expressed.

**• Section D (Practice):**

With a total of nine questions, this section evaluated dental students' practice regarding PEP. HBV vaccination, exposure to HIV/HBV risky conditions, using PEP after exposure, reasons for not using PEP, reasons for using PEP, timing to start PEP, length of PEP, completion, and reasons for discontinuing PEP were among the topics discussed.

### Scoring of knowledge, attitude, and practice

The scoring system applied in this study was according to Eticha and Gemeda [18]. Participants were considered to have "adequate knowledge" if they correctly answered at least 7 (about 75%) of the 9 equally scored knowledge questions (one point for each correct answer and zero otherwise). Similarly, participants were considered to have a "positive attitude" if their scores on the attitude questions were greater than or equal to 75% (5 out of 7 questions). Those who replied correctly to at least seven out of nine questions (about 75%) were practicing PEP.

### Bias

Selection bias was avoided by handling the questionnaire to all dental students registered in the fifth year. Information bias was avoided by providing them with explanation of the aim of the study without guiding them to the answers. Reporting bias was avoided by reporting all data assessed and avoiding selective data reporting.

### Ethical aspects

The study was carried out in accordance with ethical guidelines of Faculty of Dentistry- Cairo University. It was following the Declaration of Helsinki and approved by Research Ethics Committee, Faculty of Dentistry- Cairo University, with approval number (19.9.7). The protocol was registered in clinicaltrials.gov with an ID number NCT03893383 on 28/03/2019. During distribution of the questionnaire, dental students were provided with a full explanation concerning the aim of the study and asked them to voluntarily participate by filling the questionnaire. The written informed consent was present at the beginning of the questionnaire and the students were instructed to read the information provided before filling it. Anonymous structured questionnaire was used in this study to maintain the confidentiality of the participants.

### Statistical analysis

Fisher’s exact test was used to analyze categorial data which were presented as frequencies (n) and percentages (%). The mean and standard deviation values were used to present quantitative data. *P*-values were adjusted for multiple comparisons applying Bonferroni correction. For all tests, the significance level was set at *p* ≤ 0.05. Statistical analysis was performed with R statistical analysis software version 4.1.1 for Windows.

## Results

### Demographic data

A number of 404 students answered the questionnaire, 194 (48.0%) of which were males and 210 (52.0%) were females. The mean age of the students was (22.69 ± 1.55) years as displayed in Table 1.

**Table 1:** Showing summary statistics for demographic data

Parameter		Value
**Sex**		
**Male**	**n**	194
	**%**	48.0%
**Female**	**n**	210
	**%**	52.0%
**Age**	**Mean ± SD**	22.69 ± 1.55

### Knowledge

Majority of participants had prior knowledge regarding PEP. Among these, 29.5% reported getting information from clinical training. Participants stated that needlestick injury is the main indication for PEP (47.5%). Majority (70.4%) mentioned that PEP is preferred to be taken within an hour of exposure as revealed in Table 2. Interestingly, participants with adequate knowledge were 6 (1.5%) with a total mean knowledge score of (0.45 ± 0.50).

**Table 2:** Showing frequency and percentage (%) for answers to knowledge questions with total mean score

Question	Answers	n	%	*p*-value
**1-Have you heard about PEP?**	**Yes**	312^A^	77.2%	< 0.001*
	**No**	92^B^	22.8%	
**2-Soure of information about PEP?**	**Clinical training**	135^A^	29.5%	< 0.001*
	**Mass media**	44^BC^	9.6%	
	**Journals**	25^C^	5.5%	
	**Friends**	104^A^	22.7%	
	**Seminars**	27^C^	5.9%	
	**Textbooks**	65^B^	14.2%	
	**Others**	58^B^	12.7%	
**3-When do you think PEP should be indicated?**	**When the source patient is at high risk for HIV/H**	54^C^	12.1%	< 0.001*
	**When the patient is known to be HIV/HBV positive**	114^B^	25.4%	
	**When the HIV/HBV status of the source is unknown**	67^C^	15.0%	
	**For any needle stick injury in the workplace**	213^A^	47.5%	
**4-What is the maximum delay for PEP?**	**Within an hour**	72^BC^	17.6%	< 0.001*
	**12 h**	145^A^	35.4%	
	**24 h**	106^B^	25.9%	
	**48 h**	46^CD^	11.2%	
	**72 h**	41^D^	10.0%	
**5-What is the preferable time to take PEP?**	**Within an hour of exposure**	283^A^	70.4%	< 0.001*
	**After 6 h of exposure**	52^B^	12.9%	
	**After 12 h of exposure**	62^B^	15.4%	
	**After 72 h of exposure**	5^C^	1.2%	
**6-What is the effectiveness of PEP?**	**20–30%**	20^D^	5.0%	< 0.001*
	**30–50%**	53^C^	13.3%	
	**60–70%**	183^A^	46.0%	
	**80–100%**	118^B^	29.6%	
	**100%**	24^D^	6.0%	
**7-What is the length of time to take PEP?**	**For 28 days**	165^A^	41.4%	< 0.001*
	**For 40 days**	81^B^	20.3%	
	**For 6 months**	114^B^	28.6%	
	**For life**	39^C^	9.8%	
**8-Have you attended any training about PEP?**	**Yes**	43^B^	10.7%	< 0.001*
	**No**	358^A^	89.3%	
**9-Do you know about the PEP guidelines?**	**Yes**	84^B^	20.9%	< 0.001*
	**No**	317^A^	79.1%	
**Total mean knowledge score**	**0.45 ± 0.50**			
**Median (range)**	**0.00(1.00)**			

^*^statistically significant difference within the same question (*p* ≤ 0.05)

### Attitude

The total mean attitude score for participants was (0.70 ± 0.46). Participants with positive attitude were 122 (30.2%). Majority of participants revealed that PEP is important (94%) as shown in Table 3.

**Table 3:** Showing frequency and percentage (%) for answers to attitude questions with total mean score

Question	Answers	n	%	*p*-value
**10-Do you think PEP is important?**	**Yes**	379^A^	94.0%	< 0.001*
	**No**	6^B^	1.5%	
	**Not sure**	18^B^	4.5%	
**11-Do you believe that training of PEP is important for a behavioral change?**	**Agree**	345^A^	85.6%	< 0.001*
	**Disagree**	10^C^	2.5%	
	**Neutral**	48^B^	11.9%	
**12-Do you think there should be a PEP guideline in the workplace?**	**Agree**	281^A^	69.9%	< 0.001*
	**Strongly agree**	104^B^	25.9%	
	**Disagree**	17^C^	4.2%	
**13-Do you believe PEP reduces the likelihood of being HIV positive?**	**Yes**	259^A^	64.3%	< 0.001*
	**No**	49^C^	12.2%	
	**Not sure**	95^B^	23.6%	
**14-Do you believe PEP helps to prevent further infection?**	**Agree**	287^A^	71.2%	< 0.001*
	**Partially agree**	108^B^	26.8%	
	**Disagree**	8^C^	2.0%	
**15-What is your opinion on the saying that PEP is indicated for any type of sharp injuries?**	**Agree**	234^A^	58.1%	< 0.001*
	**Disagree**	80^B^	19.9%	
	**Not sure**	89^B^	22.1%	
**16-What is your opinion on the belief that PEP is not important if the exposure is not with patient blood of known HIV/HBV positivity?**	**Agree**	140^B^	34.7%	< 0.001*
	**Disagree**	186^A^	46.2%	
	**Not sure**	77^C^	19.1%	
**Total mean attitude score**	**0.70 ± 0.46**			
**Median (range)**	**1.00(1.00)**			

^*^statistically significant difference within the same question (*p* ≤ 0.05)

### Practice

More than half of the participants were vaccinated against HBV (71.2%). Majority of them stated that they did not take PEP after exposure (76.3%). Several participants 143 (32.5%) were not aware of the existence of PEP service and protocol as displayed in Table 4. The total mean practice score was (0.45 ± 0.50) with only 4 participants (1%) practicing PEP.

**Table 4:** Showing frequency and percentage (%) for answers to practice questions with total mean score

Question	Answers	n	%	*p*-value
**17-Have you been vaccinated against Hepatitis B?**	**Yes**	287^A^	71.2%	< 0.001*
	**No**	116^B^	28.8%	
**18-Ever, been exposed to HIV/HBV risky conditions?**	**Yes**	154^B^	38.2%	< 0.001*
	**No**	215^A^	53.3%	
	**I don’t remember**	34^C^	8.4%	
**19-Did you take PEP after exposure?**	**Yes**	92^B^	23.7%	< 0.001*
	**No**	297^A^	76.3%	
**20-Reasons for not taking PEP?**	**Unaware of the existence of PEP service and protocol**	143^A^	32.5%	
	**Lack of understanding the value of reporting exposures**	79^B^	18.0%	
	**Fear of stigma and discrimination**	30^C^	6.8%	
	**Lack of support and encouragement to report**	58^B^	13.2%	
	**PEP service is unavailable**	63^B^	14.3%	
	**Patient tested negative**	67^B^	15.2%	
**21-Reasons respondents took PEP after exposure**	**Exposure to blood from known HIV/HBV positive patients**	135^A^	32.5%	< 0.001*
	**Exposure to blood from patients with unknown HIV/HBV status**	119^A^	28.6%	
	**Injury from a sharp object**	126^A^	30.3%	
	**Contact with patient’s body fluids**	36^B^	8.7%	
**22-What time did you start taking PEP?**	**Within 1 h**	153^A^	51.9%	< 0.001*
	**After 2–6 h of exposure**	70^B^	23.7%	
	**After 6–10 h of exposure**	45^BC^	15.3%	
	**After 72 h**	27^C^	9.2%	
**23-A period of time that a respondent took PEP?**	**3 days**	136^A^	46.9%	< 0.001*
	**15 days**	75^B^	25.9%	
	**28 days**	41^C^	14.1%	
	**48 days**	38^C^	13.1%	
**24-Did you complete the prescribed drug of PEP?**	**Yes**	101^B^	34.9%	
	**No**	188^A^	65.1%	
**25-Reason for discontinuation of the drug?**	**Fear of adverse effects**	87^A^	29.5%	< 0.001*
	**Assuming that it was enough**	100^A^	33.9%	
	**Assuming that the drug was not effective**	35^B^	11.9%	
	**Patient tested negative**	73^A^	24.7%	
**Total mean practice score**	**0.45 ± 0.50**			
**Median (range)**	**0.00(1.00)**			

^*^statistically significant difference within the same question (*p* ≤ 0.05)

### Associations with gender

There was no statistically significant association between gender and all the questionnaire questions (*p* > 0.05).

## Discussion

Up to our knowledge, there was no previous study in Egypt that was carried out to assess KAP of dental students regarding PEP. Also, there is lack of data regarding PEP among dental students. Dental students included in our study were from the fifth year only to standardize the perceived knowledge and level of experience. On the other hand, the study included males and females to compare the impact of gender on their knowledge, attitude, and practice.

Our study shows that most of the participants heard about PEP, on the contrary, studies conducted in tertiary and teaching hospitals in Nigeria, Ethiopia, and Sudan revealed the opposite. This is due to the inclusion of infection control guidelines in the curriculum of our dental students in the private university. But updates, seminars or trainings on PEP and universal precaution guidelines are not routinely performed for the students. Therefore, the students lean on clinical training and their friends to learn about PEP in accordance with Agaba et al., Mathewos et al., Okoh & Saheeb and Elsadig [17, 19, 20].

We found that our students had knowledge of the preferable time for taking PEP while, Okoh & Saheeb [20] reported higher percentage than our students. But our results have higher percentage than both Elsadig [21] and Kasat el al. [22]. The disparity in the results might be attributable to differences in awareness levels among the comparable study populations or might be due to the variance level of experience and knowledge between being a postgraduate practitioner and undergraduate student which is the situation in our study.

Our study shows that less than half of the students had positive knowledge regarding the length of time of PEP. This value is low compared to the results of Mathewos et al. [17] and Okoh & Saheeb [20] on two different populations, healthcare workers in Ethiopia and dental surgeons in Nigeria respectively, while higher than results reached by Elsadig [21] among Sudanese dentists.

Majority of the dental students in our study had inappropriate knowledge about PEP for blood-borne viral infections, which is in agreement with the results of Okoh & Saheeb [20] and Elsadig [21]. Thus, knowledge of the dental students regarding PEP must be strengthened through training and education programs conducted regularly.

Our study revealed a positive attitude towards PEP for blood-borne viruses. Majority of our participants were aware of the importance of PEP on blood-borne viruses, which was comparable to the results reported by Elsadig [21]. Furthermore, this finding was lower than the that reported by Mathewos et al. [17] and Okoh & Saheeb [20], but much higher than that reported by Alenyo & Fualal [23].

Although our study showed that most of the dental students were vaccinated against HBV, the results are lower than reports from previous studies conducted by Khan et al. [6] and Shaghaghian et al. [24], as our university does not have a policy requiring students to be vaccinated against HBV.

We found that there was no gender difference regarding PEP, as both gender study the same courses and curriculum. In agreement to Khan et al. [6] who found no gender difference among Pakistanian medical students. While Wibabara et al. [25] disagreed with our findings as it was stated that there is gender differences among Ugandan medical students and females had better knowledge and vaccination status than males, which may be justified by the fact that females are generally better health seekers than males and are usually very concerned about their body and are less tolerant to changes in their health.

The strength of our study is represented in the fact that Egypt contains more than seven privately owned universities that accommodate dental schools and most of them are in Cairo implying that this study can be generalized to the whole private universities across the country. Thus, curriculum changes can be recommended to private universities to include infection control service and protocol regarding PEP. Additionally, data was collected using questionnaires without the interference of human, making it convenient and economical. All the participants were available in the same place, and majority of them kindly accepted to participate. In addition, our study deserves full attention from health policy makers to improve awareness, training, and provision of PEP to decrease the burden on the economy.

### Study limitations

There were certain limitations to this study. To begin with, dental students’ responses may differ from what they knew and practiced due to their desire to be more competent individuals of their respective profession. This might lead to the development of social desirability bias. Secondly, our study requires participants to recall and remember previous information leading to recall bias. Eventually, there were limitations during distribution of the questionnaire, as no attempt was made to follow up absent students at the day of questionnaire distribution, as well as some students refused to answer the questionnaire because they have another clinical session and no enough time, while others agreed to answer the questionnaire but did not return it back, since participation was voluntary, we could not force the students to return or answer the questionnaire.

## Conclusions

We conclude that dental students' knowledge and practice of PEP against blood-borne viral infections was unsatisfactory. Most of dental students were at risk for HIV/HBV, but only a small percentage of them utilized PEP because they were unfamiliar of the presence of PEP service and protocol. It is recommended that a structured PEP training facility with suitable guidelines should be available to maximize the employment of PEP among dental students.

## Supplementary Information

**Additional file 1.** Questionnaire.

## Data Availability

The datasets are available by the correspondent author upon reasonable request.

## Abbreviations

KAP: Knowledge, attitude, and practice
PEP: Post exposure prophylaxis
NSI: Needlestick injuries
WHO: World Health Organization
HCWs: Healthcare workers
HBV: Hepatitis B virus
HCV: Hepatitis C virus
HIV: Human immunodeficiency virus
MSA: October university for modern sciences and arts
