# Supplementary material for: Knowledge, Attitude, and Practice (KAP) of post exposure prophylaxis for fifth year dental students at a private Egyptian university: a cross-sectional study
Source: BMC Oral Health. 2023 Mar 24;23:167. doi: 10.1186/s12903-023-02890-7 (PMC10039496; doi:10.1186/s12903-023-02890-7)
Supplement: Supplementary file 1 — Additional file 1. Questionnaire. [file 12903_2023_2890_MOESM1_ESM.docx]

# Instructions

This is a cross-sectional study entitling “Knowledge- Attitude- Practice (KAP) of Post Exposure Prophylaxis for Fifth Year Dental Students of a Private Egyptian University: A Cross-sectional Study” that will be conducted to assess the knowledge, attitude, and practice of post exposure prophylaxis for fifth year dental students at this university. This study was approved by the Research Ethics Committee, Faculty of Dentistry-Cairo University with approval number (19.9.7). This self-funded study is conducted as a partial fulfillment of master’s degree in Pediatric Dentistry, Faculty of Dentistry-Cairo University.

Your responses on this questionnaire are confidential and your identity will be anonymous, and we will not ask you about your name. The information will be entered into the computer and analyzed along with all other responses. The questionnaire will take 5-10 minutes of your time, and we want to thank you for agreeing to participate in this survey. Your responses are very important to us. Your participation is voluntary, and you are free to stop filling the questionnaire at any time. For any inquiries, please contact the principle investigator of this study (Hagar Saleem) on telephone no. (01110646161) or the supervisor of this thesis (Fatma Abdelgawad) on telephone no. (01006753265). By filling this questionnaire, you are consenting participation in this study.

Please fill appropriately.

# Personal Information:

- Age:
- Gender:

# Knowledge:

Part 1: Knowledge Evaluation

## Have you heard about PEP?

- - Yes
  - No

## Source of information about PEP?

- - Clinical training
  - Mass media
  - Journals
  - Friends
  - Seminars
  - Textbooks
  - Others, state the source of information …………

## When do you think PEP should be indicated?

- - When the source patient is at high risk for HIV/HBV
  - When the patient is known to be HIV/HBV positive
  - When the HIV/HBV status of the source is unknown
  - For any needle stick injury in the workplace

## What is the maximum delay for PEP?

- - Within an hour
  - 12 h
  - 24 h
  - 48 h
  - 72 h

## What is the preferable time to take PEP?

- - Within an hour of exposure
  - After 6 h of exposure
  - After 12 h of exposure
  - After 72 h of exposure

## What is the effectiveness of PEP?

o 20-30%

o 30-50%

o 60-70%

o 80-100%

o 100%

## What is the length of time to take PEP?

- - For 28 days
  - For 40 days
  - For 6 months
  - For life

## Have you attended any training about PEP?

- - Yes
  - No

## Do you know about the PEP guidelines?

- - Yes
  - No

*Multiple answers allowed.

# Attitude:

Part 2: Attitude Evaluation

1. **Do you think PEP is important?**
   - Yes
   - No
   - Not sure
2. **Do you believe that training of PEP is important for a behavioral change?**
   - Agree
   - Disagree
   - Neutral
3. **Do you think there should be a PEP guideline in the workplace?**
   - Agree
   - Strongly agree
   - Disagree
4. **Do you believe PEP reduces the likelihood of being HIV positive?**
   - Yes
   - No
   - Not sure
5. **Do you believe PEP helps to prevent further infection?**
   - Agree
   - Partially agree
   - Disagree
6. **What is your opinion on the saying that PEP is indicated for any type of sharp injuries?**
   - Agree
   - Disagree
   - Not sure
7. **What is your opinion on the belief that PEP is not important if the exposure is not with patient blood of known HIV/HBV positivity?**
   - Agree
   - Disagree
   - Not sure

# Practice:

Part 3: Practice Evaluation

## Have you been vaccinated against Hepatitis B?

- - Yes
  - No

## Ever been exposed to HIV/HBV risky conditions?

- - Yes
  - No
  - I do not remember

## Did you take PEP after exposure?

- - Yes
  - No

## Reasons for not taking PEP?

- - Unaware of the existence of PEP service and protocol
  - Lack of understanding the value of reporting exposures
  - Fear of stigma and discrimination
  - Lack of support and encouragement to report
  - PEP service is unavailable
  - Patient tested negative

## Reasons respondents took PEP after exposure

- - Exposure to blood from known HIV/HBV positive patients
  - Exposure to blood from patients with unknown HIV/HBV status
  - Injury from a sharp object
  - Contact with patient’s body fluids

## What time did you start taking PEP?

- - Within 1 hour
  - After 2-6 hours of exposure
  - After 6-10 hours of exposure
  - After 72 hours

## 23-A period of time that a respondent took PEP?

- 3 days
- 15 days
- 28 days
- 48 days

## Did you complete the prescribed drug of PEP?

- - Yes
  - No

## Reason for discontinuation of the drug?

- - Fear of adverse effects
  - Assuming that it was enough
  - Assuming that the drug was not effective
  - Patient tested negative

*Multiple answers allowed.

*Risky conditions: blood, patient’s body fluids, needle prick/sharps injury at workplace.
